# Microwave properties of the single-layer periodic structure composites composed of ethylene-vinyl acetate and polycrystalline iron fibers

**DOI:** 10.1038/s41598-017-11884-9

**Published:** 2017-09-12

**Authors:** Zhibo Guo, Hailong Huang, Ding Xie, Hui Xia

**Affiliations:** School of physics and electronics, Central South University, Changsha, Hunan 410083 People’s Republic of China

## Abstract

A single-layer microwave absorbing structure composed of the ethylene-vinyl acetate (EVA) powders and polycrystalline iron fibers (PIFs) with thickness of 2 mm, which has a periodic array of circular hole, is designed and fabricated using the mechanical method. We show that the reflection loss (RL) can be easily adjusted by changing the geometric parameters. The maximum RL is 18.7 dB at 15 GHz, and the effective absorption bandwidth of 1.7 GHz for the diameter of circular hole is 10 mm, and enhanced to be 23.7 dB at 15.1 GHz with the corresponding bandwidth of 7.2 GHz when the diameter decreases to 5 mm. The measured absorption of the composite is in good accordance with the simulation results. Furthermore, the possible absorption mechanism of the composite has been discussed. Our results illustrate that the integration of frequency selective surface (FSS) with traditional PIFs can achieve a wide frequency range of a strong absorption.

## Introduction

Microwave absorbing materials (MAMs) are extensively utilized to absorb electromagnetic wave effectively and convert electromagnetic energy into heat or make electromagnetic wave disappear by interference^1^. Traditional MAMs such as various shapes of spongy absorbers in darkroom and stealthy coating for aircraft have been well developed^2–8^. In past decades, the composites containing different kinds of micro or nano components for microwave absorption are fabricated and tested in laboratories^9–12^. However for the traditional MAMs or other novel MAMs, such as carbon black, ferrites and metals, they all still have some disadvantages like high density, narrow band and low absorption. These disadvantages will hamper their potential applications in cloaking, lightweight absorbers. Therefore to develop more advanced MAMs with low density, broad and adjustable absorption band are in urgent.

A frequency selective surface (FSS) is a two-dimensional planar periodic array that can be used to reflect or transmit electromagnetic waves selectively^13^. Traditional metallic FSSs are not suitable to fabricate MAMs due to low absorption at its resonant frequency, so a bonding is needed to attach metallic FSS to the dielectric slabs to make effective MAMs, but the increased weight and thickness will also hamper their potential applications. Then the FSSs made of polymer matrix composite materials have been proposed. For example, Huynen. I *et al*. fabricated the lightweight MAMs, which are composed of a metallic honeycomb and carbon nanotube-reinforced polymer foam. The results show that the enhanced absorption over a wide frequency range can be obtained^14^. Chen *et al*. reported a lightweight microwave absorbing structure with two dimensional composite lattice cores, which can be regarded as an effective absorber with a wider absorption band^15^. Kim *et al*. prepared the MAM composed of FSS screens and E-glass/epoxy composite using the printed circuit board method, which showed the good microwave absorption properties^16^. Khurram *et al*. prepared high performance MAMs by filling a thermoplastic resin into a honeycomb cores coated with graphite and multiwalled carbon nanotubes^17^. Recently, many researchers have indicated that soft magnetic ferrite and ferroelectric substances are promising MAMs. Liu *et al*. have synthesized Fe nanowires/epoxy resin composites, and they can provide good electromagnetic wave absorption^18^. Dosoudil *et al*. fabricated the metal alloy/spinel ferrite/polymer composites, and showed an excellent electromagnetic wave absorption in the quasi-microwave band^19^. However, the above methods are unsuitable for the large-scale production at low cost. The single-layer structure is easy to implement in the demand of large-production^20, 21^, it is expected that the single-layer polymer composites based on the combination of polycrystalline iron fibers (PIFs) with a FSS structure can be a new kind of the MAMs.

In this paper, a single-layer MAM composed of PIFs and flexible ethylene-vinyl acetate (EVA) matrix along with a periodic arrangement of circular hole is designed and fabricated by a simple mechanical method. The effects of the weight ratio of EVA to PIFs and geometric parameters on absorption of the composites are studied in the range of 4–18 GHz in experiment. The Finite Element Method (FEM) numerical simulation is used to investigate the absorption. By comparing between the simulation and measurement, the effective absorption band is in excellent coincidence. The absorption mechanism of the improved MAM is discussed. The results illustrate that the integration of FSS with traditional EVA/PIFs can achieve a strong absorption in a wide frequency range, and the maximum absorption can be easily tuned by varying the geometric parameters of the MAMs.

## Experiments

### Materials

The PIFs (in diameter of about 15 μm, in length of about 20–30 μm) are purchased from Jiangyou nuclear nano materials Co. Ltd, China. Tributyl phosphate (TBP, 98%) is obtained from Hunan Huifeng Reagent Co. Ltd, China. The EVA is obtained from AkzoNobel. Specialty Chemicals (Shanghai) Co. Ltd, China. Deionized water obtained from a Milli-Q system (Millipore, Bedford, MA) is used.

### The preparation of MAMs

**Figure 1:**
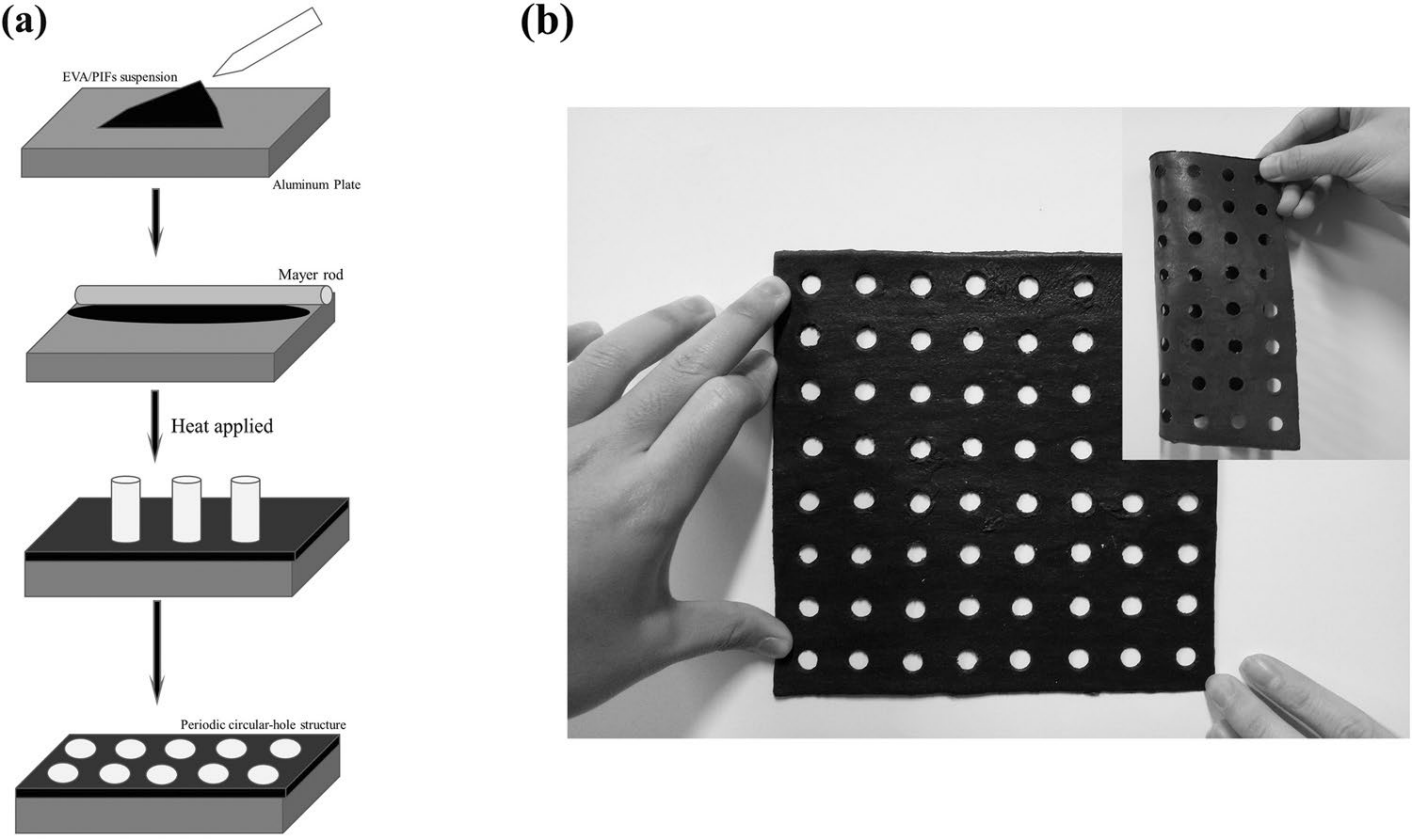
(**a**) The manufacturing process of as-prepared MA; (**b**) Optical image of the MA, the inset in Fig. 1b represents the sample’s good flexibility performance.

The EVA powders and PIFs (in a weight ratio of EVA to PIFs is 2:1) are dissolved in the Tributyl phosphate (TBP) solvent, the content of the mixture powders is 75%. After being stirred for 24 h, and the homogeneous suspension is smeared onto an aluminum plate layer by layer using the wire-wound rod coating method, and then immediately put in the vacuum oven for 2 h at 80 °C to form the MAM. Then the product is cooled at room temperature for further use. The sample is shaped into the configuration of the circular hole using the hole punch. The manufacturing process is shown by Fig. 1(a). Figure 1(b) shows the manufactured samples for absorption testing, and the inset in Fig. 1(b) represents the good flexibility performance of the sample. The dimension of the sample is 180 mm × 180 mm × 2 mm. The sample without periodic structure is also manufactured as reference. Table 1 lists the fabricated composite panels with different diameters of the circular hole at same thickness of 2 mm. In this work, the geometric parameters of all samples are chosen as follows: 5 mm and 10 mm are selected as the diameter of the circular-hole and 1.5 mm is set as the distance between adjacent holes.

**Table 1:** The fabricated composite panels with different diameter of circular hole.

Code name	Composite panels	The diameter of circular hole (mm)	Thickness (mm)
A	Pure EVA	0 (no circular hole)	2
B		10	2
C	EVA / PIFs	0 (no circular hole)	2
D		10	2
E		5	2

### Characterization

The reflection loss (RL) of the samples in the 4–18 GHz frequency range is measured by a free-space method with an NRL Arch reflectivity test setup. The scattering parameter (*S*_11_) of the panels is measured by using an aluminum plate as a perfect reflector. The morphology of the specimens is carried out with field-emission scanning electron microscopy (FESEM, Nova NanoSEM230) operated at an accelerating voltage of 10 kV after sputter-coating specimens with platinum. The samples for measuring electromagnetic parameters are prepared by dispersing the EVA/PIFs composites powders in paraffin wax. The volume fraction of the powders is 70%. The powder/wax composites are die-pressed to form cylindrical toroidal specimens with 5.0 mm outer diameter, 2.0 mm inner diameter, and 2 mm thickness. The complex effective permittivity and complex effective permeability can be obtained according to the NRW algorithm^22^. The samples are washed using distilled water several times, and dried at 60 °C for 24 h before characterization.

### Numerical calculation

The FEM solution is employed in the numerical study^23^. The analysis model of the sample is constructed including all the components of practical structures. The composite structure is embedded within vacuum space, which is truncated in three-dimensional space by an absorbing boundary known as the perfectly match layers (PMLs) to emulate the infinite space. The adaptive meshing technique is used to automatically refine the mesh in which large computational errors are found. A convergence condition is predefined to terminate the simulation automatically.

**Figure 2:**
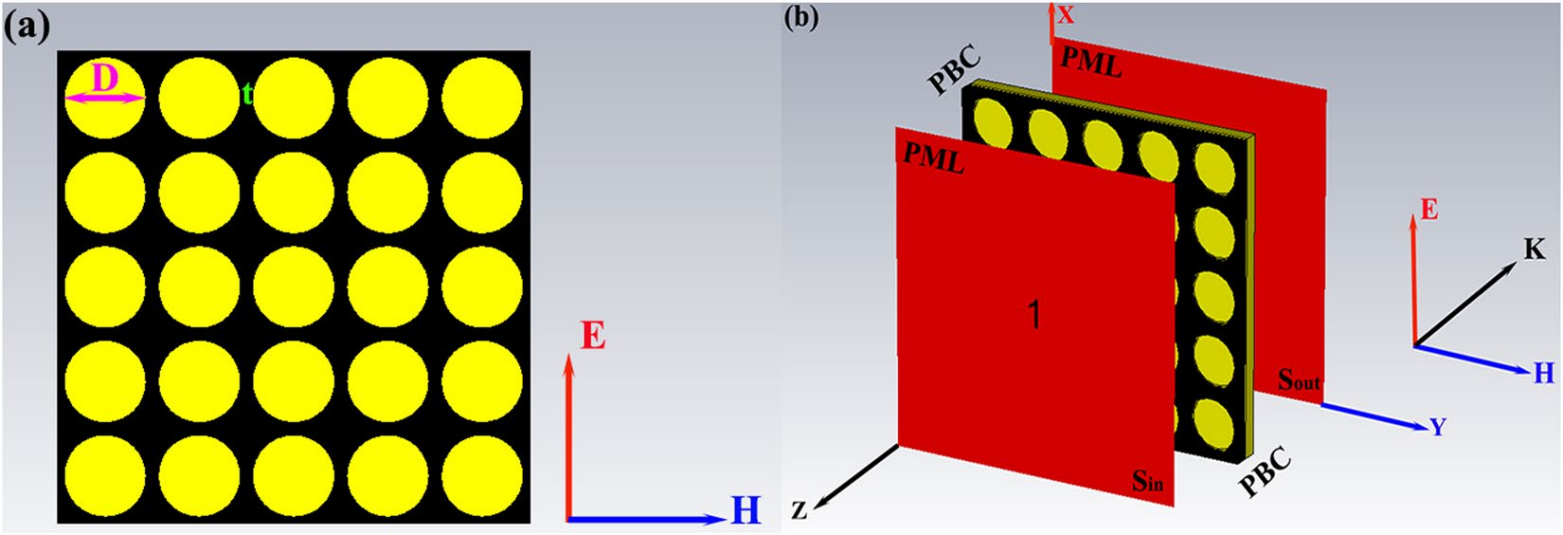
(**a**) The vertical view of numerical model, the diameter of circular hole is *D* and distance between adjacent holes is *t*; (**b**) the periodic or lined boundary conditions are applied to the model.

Figure 2(a) shows the vertical view of the numerical model, in which an aluminum plate is placed as a perfect reflector. The diameter of circular hole is *D* and the distance between adjacent holes is *t*. A plane wave with the electric field ***E*** parallel to the layer and wave vector ***K*** perpendicular to the layer surface illuminates the structure at normal incidence. The PML boundary conditions are imposed on both surfaces that are perpendicular to the wave vector, as shown in Fig. 2(b).

## Results and Discussion

**Figure 3:**
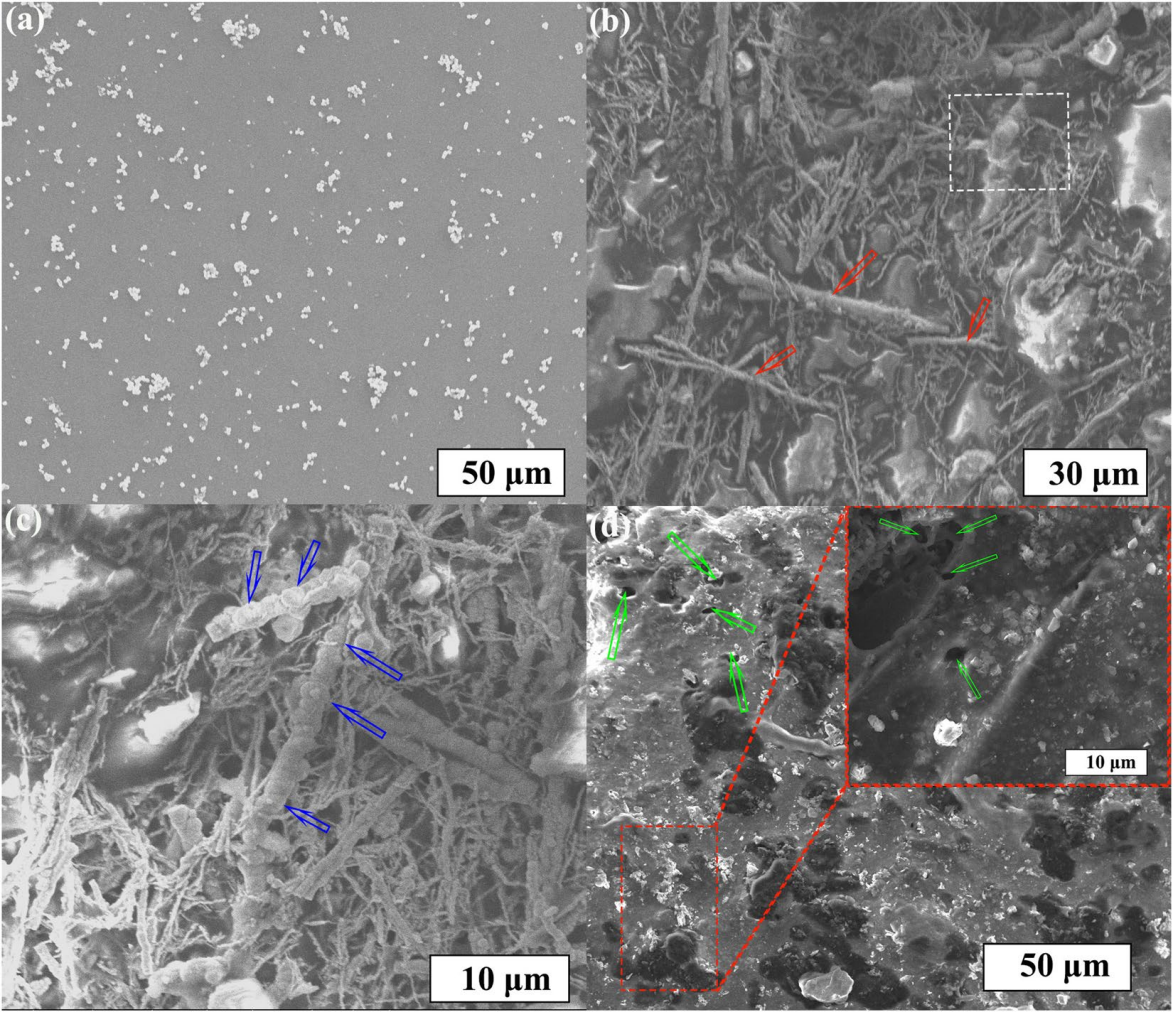
SEM image of (**a**) EVA powders. (**b**) EVA/PIFs composites in low-magnification and (**c**) high-magnification, respectively (**d**) the surface of the composites, inset image represents the surface morphology indicated by a red dotted rectangle at high magnification.

Figure 3(a) shows the SEM image of the pure EVA powder. Most of the particles are in the range of 4–7 μm. Figure 3(b) shows a typical SEM image of the prepared single-layer EVA/PIFs composites. It can be found that the distribution of the PIFs is uniform, and some fibers (indicated by red arrow) will become oriented to some extent due to the pressure loaded during the fabrication process. Figure 3(c) shows the composites under a magnified SEM image in the selected squared area of Fig. 3(b), the surface of PIFs is coated by EVA (indicated by blue arrow), which avoid the oxidation of the PIFs. Meanwhile, this hierarchical wire-like structure and good dispersion of the fibers will generate more heterogenic interfaces, and that is good for the generation of space charge polarizations in the composite and also would enhance the absorption properties, which is proved by other group^24^. Figure 3(d) shows the SEM picture of the surface of the single-layer EVA/PIFs composites. It is clearly seen that some voids (indicated by green arrow) can be found on the surface, which are helpful for reducing the mismatched impedance between the free space and composites, resulting in the excellent absorption properties at the resonance frequency, the inset image in Fig. 3(d) demonstrates that the PIFs are covered with the EVA polymer layer.

**Figure 4:**
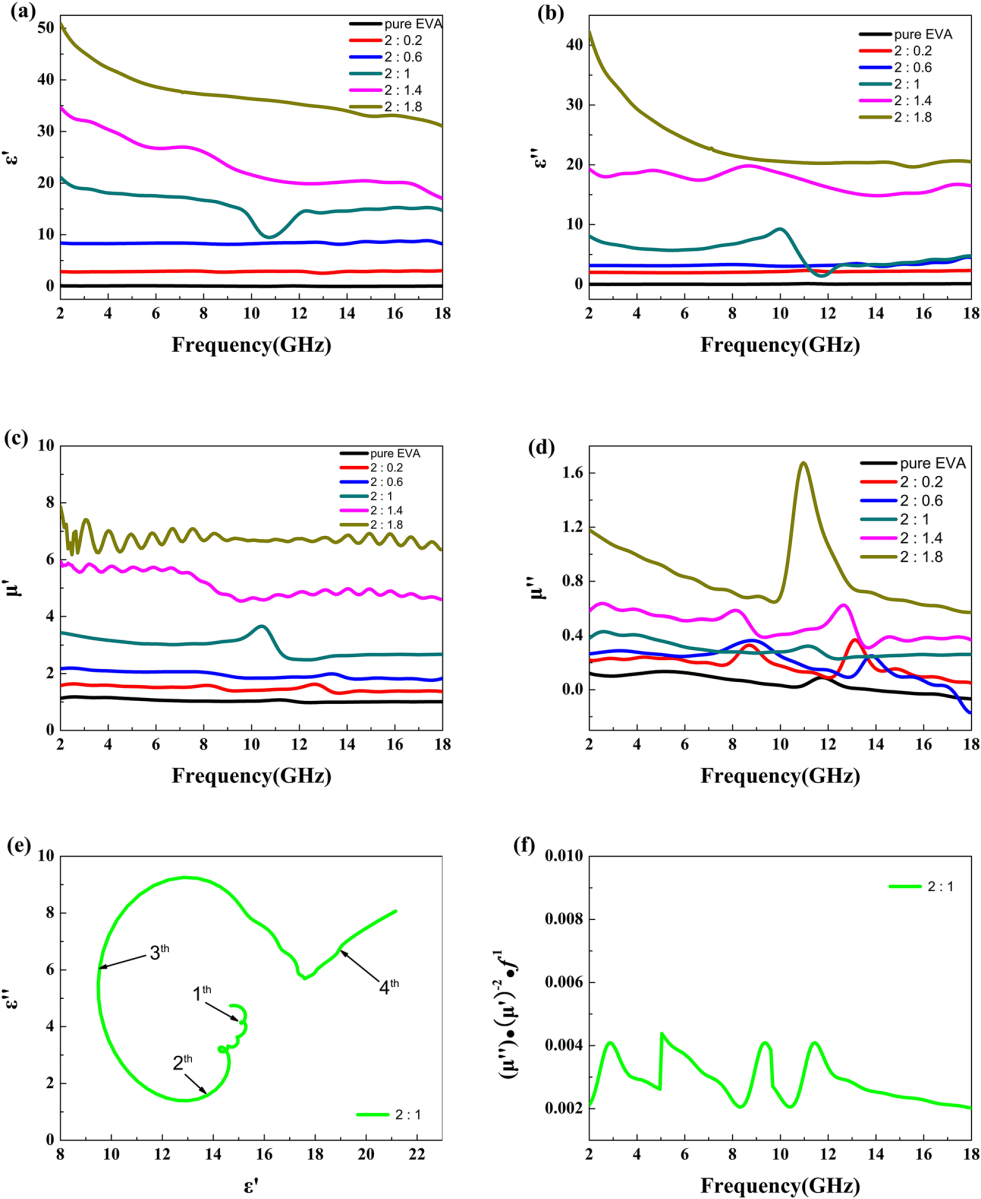
(**a**) and (**b**) the complex permittivity of the pure EVA and EVA/PIFs composites. (**c**) and (**d**) the complex permeability of the pure EVA and EVA/PIFs composites. (**e**) Typical Cole-Cole semicircles and (**f**) current data of EVA/PIFs with different weight ratio of EVA to PIFs.

The enhanced absorption performance of the EVA/PIFs composites may be mainly attributed to the improved dielectric and magnetic properties. EVA is used as a polymer matrix due to its chemical structure, which gives the molecular chain high flexibility and polarizable characteristics, and PIFs act as the absorbing medium. As shown in Fig. 4(a) and (b), the values of both *ε*′ and *ε*′′ of the pure EVA powders were almost independent of the frequency. For pure EVA, the values of the *ε*′′ were very close to zero, which demonstrated the poor dielectric loss of pure EVA. For the EVA/PIFs composites, the values of both *ε*′ and *ε*′′ displayed a certain degree of fluctuation in the frequency range of 2–18 GHz, which became increasingly pronounced with an increase in the content of PIFs. According to the free electron theory, a high electrical conductivity is favorable for a high value of complex permittivity. Thus these increases are mainly due to the high content of the PIFs. Generally, the values of *ε*′ of the EVA/PIFs (in weight ratio of 2: 1) gradually decreased with an increase in frequency. The values of *ε*′′ have some obvious resonances in 12–18 GHz, and this may have a better microwave absorption performance in the corresponding frequency range.

Generally speaking, the Debye dipolar relaxation, which can be described by the Cole-Cole semicircle, probably has a great influence on the absorption behaviors. According to Debye dipolar relaxation, the relationship between *ε*′ and *ε*′′ can be expressed by the following expression^25^:

1 $${(\varepsilon ^{\prime} -\frac{{\varepsilon }_{s}+{\varepsilon }_{\infty }}{2})}^{2}+{(\varepsilon ^{\prime\prime} )}^{2}={(\frac{{\varepsilon }_{s}-{\varepsilon }_{\infty }}{2})}^{2}$$

Where *ε*_s_ and *ε*_∞_ are the static permittivity and relative dielectric permittivity at the high-frequency limit, respectively. From Eq. (1), it is easy to see that the plot of *ε*′ versus *ε*′′ would be a single semicircle, generally regarded as the Cole-Cole semicircle. Each semicircle corresponds to one Debye relaxation process. In the present work, four semicircles were found from the curve of the EVA/PIFs (in weight ratio of 2: 1) in Fig. 4(e). The presence of three standard semicircles, which is plotted based on the data obtained from the range of 2–18 GHz, suggests that Debye dipolar relaxation is the main dielectric loss mechanism in this frequency domain. The other distorted semicircles indicate other mechanisms, such as the Maxwell-Wagner relaxation, electron/ion polarization, and interfacial polarization also exist in addition to the Debye relaxation^26–28^. Therefore, these multiple dielectric losses is helpful for the enhancement of absorption properties.

The parameters of the relative complex permeability of the pure EVA and EVA/PIFs are shown in Fig. 4(c) and (d), respectively. The real part of permeability (*μ*′) values of the EVA/PIFs displays a gradual increasing trend with increasing weight ratio of EVA to PIFs. While those of the weight ratio smaller than 2: 1 exhibit some fluctuation in the investigated frequency region. The imaginary part of permittivity (*μ*′′) values of the EVA/PIFs show the same trends with those of the real part of permeability, and a higher *μ*′′ of the EVA/PIFs shows a higher attenuation ability in terms of magnetic loss mechanism includes hysteresis loss, electrical current loss and residual loss, such as domain resonance, natural resonance, and exchange resonance. However, the hysteresis loss was proportional to the area of the hysteresis loop and was almost negligible in a weak field^29^. In addition, the magnetic loss may not have originated from domain wall resonance, which usually occurs in the megahertz frequency range. Moreover, for spinel ferrites, exchange resonance also makes little contribution to the magnetic loss in the high frequency range^30^. And the natural resonance can be also ignored due to the actual sample thickness (2 mm) is much smaller than the calculated minimum thickness (3–4 mm) by the wavelength equations^27^:

2 $$C=\lambda f$$

3 $$d=\frac{n\lambda }{2}\,(n=1,\,2,\,3,\ldots \ldots )$$

Thus the dominated mechanism is electrical conductance loss in the microwave frequency band.

According to skin-effect criterion, the electrical conductance loss can be evaluated by the following equation^31^:

4 $$\mu ^{\prime\prime} {(\mu ^{\prime} )}^{-2}{f}^{-1}=2\pi {\mu }_{0}\sigma {d}^{2}/3$$

Where *μ*_0_ is the vacuum permeability, *σ* is the electronic conductivity and *d* is the sample thickness. If magnetic loss mechanism solely originates from electrical conductance loss effect, the values of $\mu ^{\prime\prime} {(\mu ^{\prime} )}^{-2}{f}^{-1}$ will keep constant with changing *f*. As seen from Fig. 4(f), these values are nearly unchanged (0.002–0.004) for the EVA/PIFs (in the weight ratio of 2: 1) in the whole frequency range, indicating that magnetic loss is mainly caused by electrical current effect.

To study the absorption properties of EVA/PIFs composites, the reflection loss (RL) is calculated using the transmission line theory method with the following formula^32^:

5 $$\,{Z}_{in}={Z}_{0}{({\mu }_{r}/{\varepsilon }_{r})}^{1/2}\,\tanh [j(2\pi fd/c){({\mu }_{r}{\varepsilon }_{r})}^{1/2}]$$

6 $${\rm{RL}}=20\,\mathrm{log}|({Z}_{in}-{Z}_{0})/({Z}_{in}+{Z}_{0})|$$

where *Z*_in_ is the input impedance of the absorber, *Z*_0_ is the impedance of free space, *μ*_r_ is the relative complex permeability, *ε*_r_ is the complex permittivity, *f* is the frequency of microwaves, *d* is the thickness of the absorber, and *c* is the velocity of light, and a RL value lower than −10 dB which means that 90% of incident microwaves can be absorbed.

**Figure 5:**
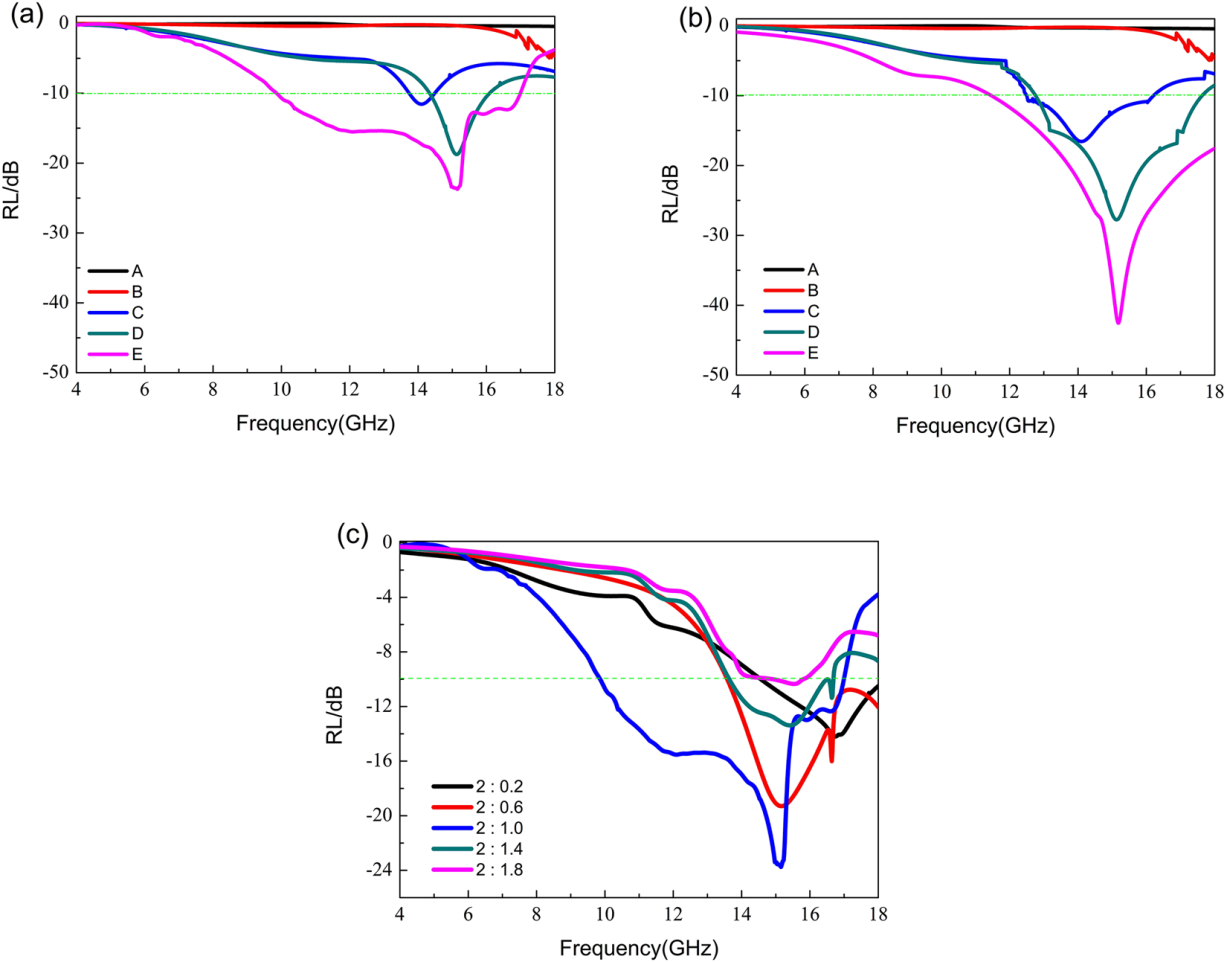
(**a**) The measured reflection loss of the MA versus frequency. (**b**) The RL calculated by FEM simulation. (**c**) The absorption curves of the composites in different weight ratio of EVA to PIFs.

Figure 5(a) shows the measured RL versus frequency for different MAMs listed in Table 1 in the range from 4 to 18 GHz. It is clearly seen that the absorption of sample A and B are very weak. The results show that the polymer EVA has no significant effect on attenuating microwave radiation. In contrast, the other samples have strong absorption towards microwave radiation, as shown in Fig. 5(a). The maximum absorption of the sample C is 11.6 dB at 14.0 GHz with the thickness of 2 mm, and the effective absorption frequency ranges from 13.7 to 14.4 GHz. The maximum absorption of sample D is 18.7 dB at 15 GHz, and the effective absorption frequency ranges from 14.3 to 16.0 GHz. The best result can be obtained in sample E for *D* = 5 mm. Its maximum absorption reaches 23.7 dB at 15.1 GHz at same thickness of 2 mm, and the frequency ranges in which effective absorption less than −10 dB is from 9.8 to 17.0 GHz. The strong absorption frequency ranges from 9.8 to 16.9 GHz for the sample D, and shifts to 12.4–18 GH for the sample E. The shifted absorption frequency is probably due to the different diameter of the circular hole, which is similar to the ref. 33. Figure 5(b) shows the RL of EVA/PIFs composites with a thickness of 2 mm calculated by the FEM. As shown in the Fig. 5(a) and (b), the calculation results are in agreement with that of the experiments. For the samples C and D, the maximum absorption values calculated by the FEM are 16.5 dB and 27.7 dB, which are larger than the experimental values of 11.6 dB and 18.7 dB, respectively. For sample E, the maximum absorption value predicted by the FEM is 42.5 dB, which is larger than the experimental values of 23.7 dB. The reasons for the differences between the calculated and experimental results are that the finite EVA/PIFs composites are supposed to be infinite in the FEM, and the calculated parameters may be different from the experimental procedure as well as the measurement. The weight ratio of EVA to PIFs, which can also affect the matched impedance between absorber and free space, should be carefully considered in the practical application. Figure 5(c) shows the absorption curves of the composites with different weight ratio of EVA to PIFs, in which *D* = 5 mm, *t* = 1.5 mm and the thickness is 2 mm. The absorption properties of the prepared EVA/PIFs are summarized in Table 2. From Fig. 5(c), it is observed that the absorption will be affected by the weight ratio of the EVA to PIFs. For example, when the weight ratio of EVA to PIFs is lower than 2, the absorption is effectively enhanced with the increase weight ratio of EVA to PIFs. But in the case of weight ratio is larger than 2, the absorption of corresponding composites will decrease as the increased weight ratio of EVA to PIFs. This means that the absorption performance of the materials is strongly depending on the weight ratio of EVA to PIFs. In the experiment, the observed increase in the imaginary part of the electromagnetic parameters (as shown in Fig. 4(b) and (d)) of the EVA/PIFs will cause high electrical conductivity so that the skin depth is very small and nearly most of waves would be reflected on the surface of the absorbers^8, 34^. This result shows that the parameter in the weight ratio of EVA to PIFs is chosen reasonably. In Table 3, the microwave absorption performance of the present EVA/PIFs composite is compared with that of other magnetic materials. The results indicate that our composite exhibit significantly improved absorption properties in a thinner thickness.

**Table 2:** Absorption properties of the EVA/PIFs.

The weight ratio of EVA to polycrystalline iron fibers	△*f* (GHz)	*RL*_min_ (dB) (*f*_im_(GHz))
2: 0.2	3.4(14.6–18.0)	−14.2(16.7)
2: 0.6	4.4(13.6–18.0)	−19.2(15.1)
2: 1.0	7.2(9.8–17.0)	−23.7(15.1)
2: 1.4	3.0(13.6–16.6)	−13.3(15.4)
2 :1.8	0.7(14.9–15.8)	−10.3(15.5)

△*f*, *RL*_min_, *f*_im_ represent bandwidth of reflection loss below −10 dB, minimum reflection loss and frequency of *RL*_min_, respectively.

**Table 3:** Comparison of the absorption performance of EVA/PIFs composite with those of other magnetic materials.

Materials	Maximum RL value (dB)	Absorption bandwidth (GHz)	Thickness (mm)	Ref.
G/Fe_50_Ni_50_	23.9	4.3	3.0	35
G/Ni	13.0	3.6	2.0	36
G/Fe_3_O_4_	28.0	1.2	1.5	37
G/porous Fe_3_O_4_	20.0	4.5	2.0	38
HGS@Fe_3_O_4_@RGO	15.8	3.6	2.5	39
EVA/PIFs	23.7	7.2	2.0	This work

**Figure 6:**
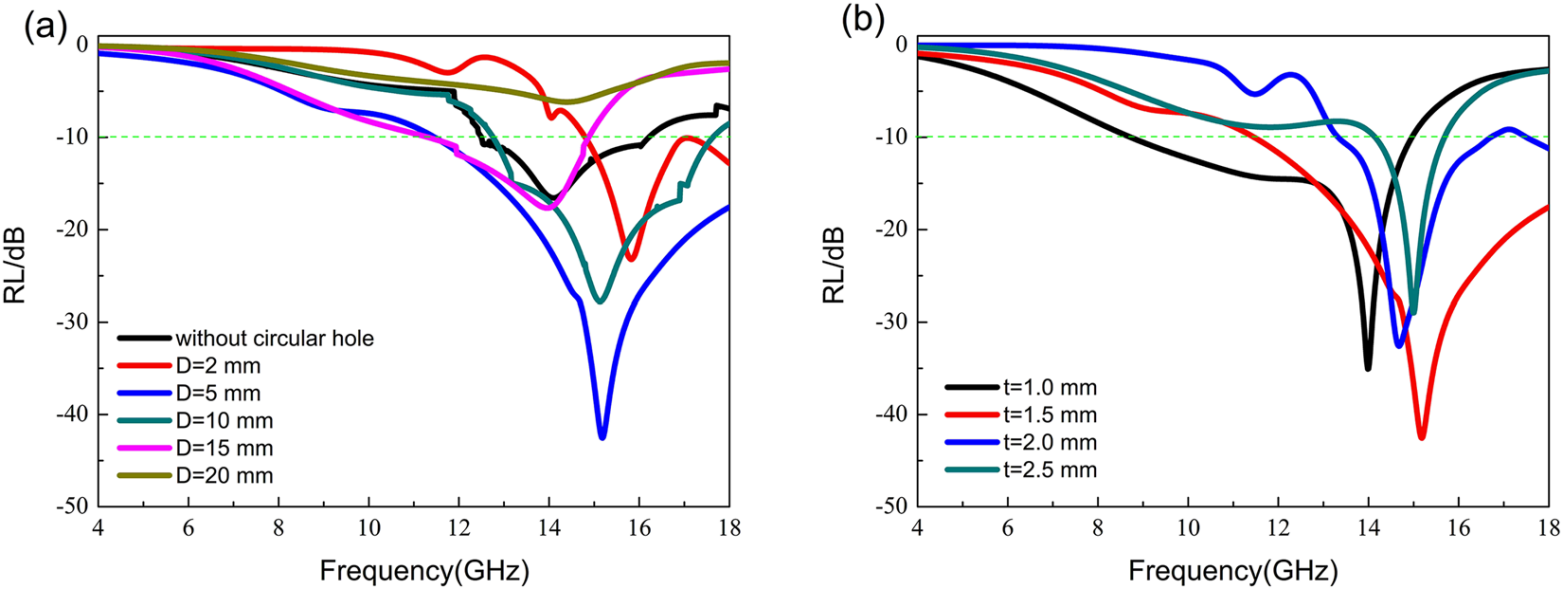
The calculated RL of the MA with (**a**) the different diameters of the circular hole *D*, and the *t* is fixed at 1.5 mm (**b**) the different distance between adjacent circular holes *t*, the *D* is fixed at 5 mm.

To further investigate the influence of the geometric parameters on the performance of the MAM, the calculated RL with different *D* and *t* are shown in Fig. 6(a) and (b), respectively. With no circular hole, the maximum absorption of the composites approaches to 16.5 dB. As shown in Fig. 6(a), it is found that the *RL* increases with the increased diameters of the circular hole when *D* < 10 mm. For the case of *D* = 2 mm, the maximum absorption can reach to be 23.2 dB, and it increases to be 42.5 dB for *D* = 5 mm. But the RL decreases with the increased *D* in the case of *D* > 10 mm. When *D* = 10 mm, the maximum absorption is 27.7 dB, and it decreases to be 6.1 dB when *D* = 20 mm. Figure 6(b) shows the absorption properties of the composites with different distance *t*. It can be seen that the absorption is enhanced with *t* increases for the case of *t* < 1.5 mm. For *t* > 1.5 mm, the absorption is decreased with the increased *t*. The results show that the choices of geometric parameters in the sample D and E are reasonable, and the influence of the diameters *D* on absorption performance is stronger than that of the distance *t*.

**Figure 7:**
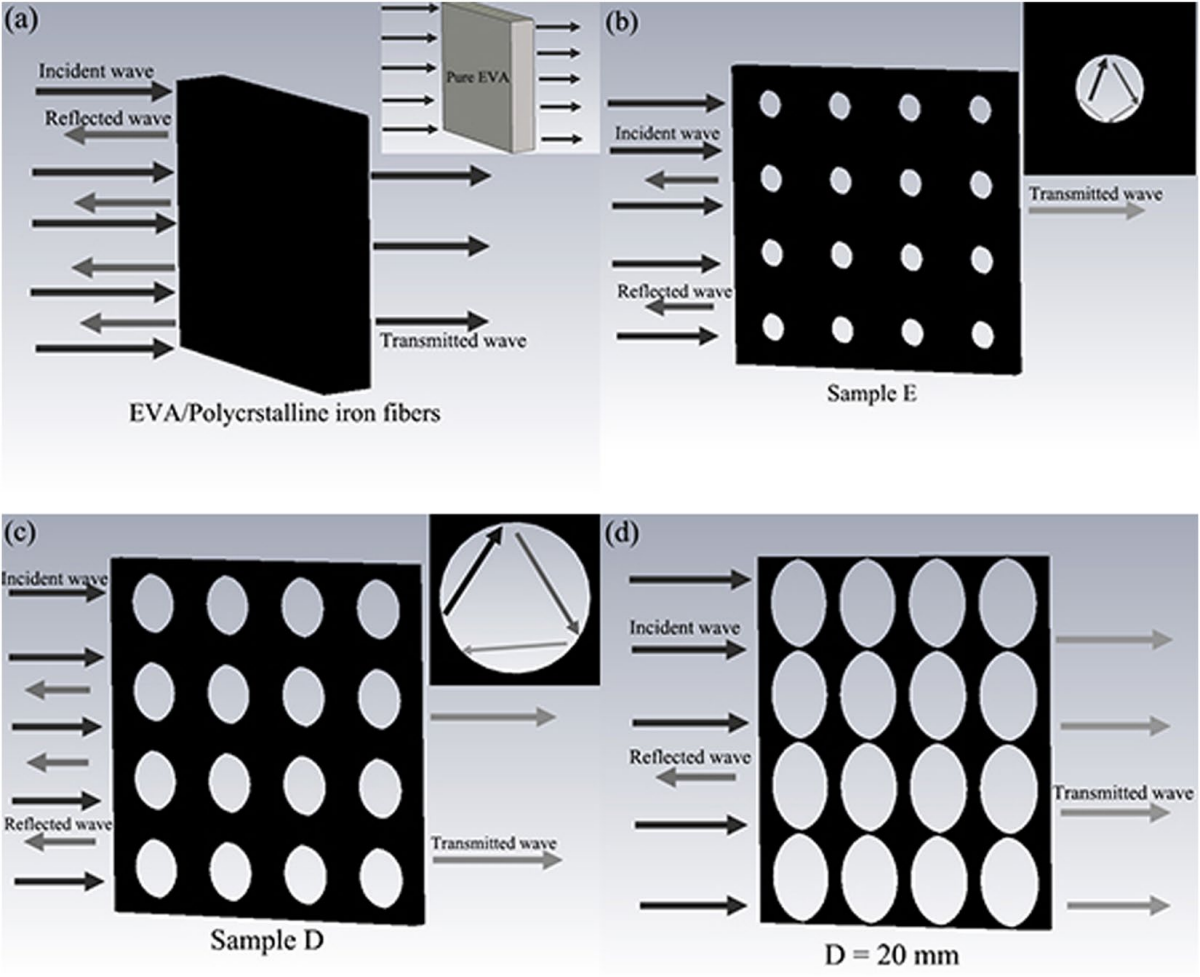
Schematic descriptions of the MA mechanism of (**a**) EVA/PIFs, the inset represents the pure EVA panels. (**b**) Sample E, the inset represents the microwave reflected by a unit cell. (**c**) Sample D, the inset represents the microwave reflected by a unit cell. (**d**) MA with the diameter of the circular hole is *D* = 20 mm.

To better understand the impact of the circular hole on the absorption mechanism, some aspects of the characteristics of the absorber need to be understood. It is shown that the absorption of sample C is better than that of pure EVA due to the magnetic loss of the PIFs, as shown in Fig. 7(a). From Fig. 7(b) and (c), it is found that the enhanced absorption can be simply realized by the presence of the periodic array of circular holes because of the reduced impedance mismatch between the absorber and free space. Figure 7(d) shows the absorption mechanism of composites with D = 20 mm. It is found that most waves can penetrate through the samples, and leading to a lower absorption in the absorber. When an electromagnetic wave is incident on the surface of the absorber, the wave typically will be reflected due to the impedance mismatch exists in the absorber, however, some voids existing on the surface of the composites and circular hole can reduce the impedance mismatch, which enables wave to penetrate the absorber more easily. The behavior is different from that of absorption with a flat surface, and it greatly reduces the intensity of the reflected wave in the opposite direction of the incident wave. The scattering causes the electromagnetic wave to be sufficiently reflected and absorbed within the circular hole. The content of PIFs decreases with the diameters of the circular hole increasing so that the electromagnetic wave can fully transparent to the MAM. In other words, larger air holes will bring the impedance of the EVA/PIFs much closer to that of free space, and resulting in a lower absorption than other composites in smaller air holes. On the contrary, the smaller air holes will make the mismatched impedance between EVA/PIFs and air more obvious, and leading to a larger reflection. Thus the impact of air holes is obvious since there is always a trade-off between the impedance match and the filling ratio of absorbing materials.

## Conclusions

We designed and fabricated the lightweight single-layer MAMs (2 mm in thickness) composed of a periodic arrangement of circular hole (5 mm in diameter). The effects of the geometric parameters and weight ratio of EVA to PIFs on the absorption of the composites are investigated by both experiment and simulation in detail. The simulation results agree well with the measured ones in the whole frequency region. The experimental results show that when the weight ratio of EVA to PIFs is 2:1, the maximum RL is 18.7 dB at 15 GHz, and the effective absorption frequency ranges from 14.3 to 16.0 GHz when the diameter of circular hole is 10 mm, and it enhanced to be 23.7 dB at 15.1 GHz, with the effective absorption frequency ranges from 9.8 to 17.0 GHz when the diameter of the circular hole decreases to 5 mm. The simulation results show that the influence of the diameter of circular hole on absorption is much stronger than that of distance between adjacent holes. The absorption mechanism of the composite has been discussed. As a result, the designed single-layer MAMs may have potential applications in cloaking and stealth radomes.
